# The effect of modified donanemab titration on amyloid-related imaging abnormalities with edema/effusions and amyloid reduction: 18-month results from TRAILBLAZER-ALZ 6

**DOI:** 10.1016/j.tjpad.2025.100266

**Published:** 2025-07-05

**Authors:** Hong Wang, Emel Serap Monkul Nery, Paul Ardayfio, Rashna Khanna, Diana Otero Svaldi, Sergey Shcherbinin, Wen Xu, Scott W. Andersen, Paula M. Hauck, Dawn A. Brooks, Emily C. Collins, Stephen Salloway, Mark A. Mintun, John R. Sims

**Affiliations:** a Eli Lilly and Company, Indianapolis, IN, USA; b Butler Hospital, and Department of Neurology and Department of Psychiatry, Warren Alpert Medical School of Brown University, Providence, RI, USA

**Keywords:** Alzheimer’s disease, Amyloid, amyloid-related imaging abnormalities (ARIA), Donanemab, Titration

## Abstract

The TRAILBLAZER-ALZ 6 study (NCT05738486) evaluated the effect of different donanemab dosing regimens on amyloid-related imaging abnormalities with edema/sulcal effusions (ARIA-E). The modified titration arm met the primary outcome and significantly reduced ARIA-E frequency compared with the standard dosing while maintaining a similar pharmacodynamic effect on amyloid reduction at 24 weeks. Primary outcome and 52-week data were previously published. Completed study results at 76 weeks are reported here. ARIA-E frequencies were 15.6 % and 24.2 % in the modified titration and standard arms, respectively. ARIA-E radiographic severity was significantly lower (*p* = 0.015) with modified titration than with standard dosing. Additionally, symptomatic ARIA-E frequency was lower with modified titration versus standard dosing (2.8 % vs 4.8 %). The frequency of serious adverse events was comparable between the modified titration and standard dosing arms. A more gradual titration of donanemab dosing significantly reduced ARIA-E risk versus standard dosing.

**Clinicaltrials.gov:**

NCT05738486

## 1 Introduction

Donanemab has been approved in multiple countries for the treatment of early symptomatic Alzheimer’s disease (AD) with the standard dosing regimen of 700 mg every 4 weeks for the first 3 doses followed by 1400 mg every 4 weeks. Amyloid-related imaging abnormalities (ARIA) with edema/effusions (ARIA-E) can have adverse outcomes and are associated with amyloid-targeting therapies such as donanemab [1].

The TRAILBLAZER-ALZ 6 study investigated the effects of different donanemab dosing regimens on the frequency and severity of ARIA-E and the extent of amyloid lowering in participants with early symptomatic AD.

As ARIA-E is more likely to occur within 6 months after donanemab initiation, the timing and dosage of initial infusions are important considerations in ARIA-E reduction [[1], [2], [3]]. Given that evidence obtained in the TRAILBLAZER-ALZ and TRAILBLAZER ALZ 2 trials demonstrated that titration decreased ARIA-E, the modified titration arm extended titration further than the standard dosing regimen. Dose skipping was proposed to allow time for tissue recovery and adaptation (vascular healing). The Cmax hypothesis was suggested as a driver of ARIA by others in the field at the time of study design [[4], [5], [6]]. Selection of the alternative dosing regimens was also based on safety, pharmacokinetic, pharmacodynamic, and efficacy modeling from phase 1 through 3 donanemab clinical studies [7]. All dosing regimens were expected to result in equivalent reduction of amyloid plaques.

We previously reported that the modified titration arm (once-monthly dosing of 350 mg, 700 mg, 1050 mg, and then 1400 mg thereafter) met the primary objective at week 24 demonstrating a 41 % relative risk reduction (RRR) of ARIA-E compared with the standard dosing arm, with a 94 % probability that the RRR was at least 20 % [8]. Here, we report the 76-week results of the TRAILBLAZER-ALZ 6 study that confirm the primary endpoint results, demonstrating that the modified titration regimen, compared with the standard regimen, significantly reduces ARIA-E risk and severity while maintaining comparable pharmacodynamic effects through 76 weeks of donanemab treatment.

## 2 Methods

### 2.1 Study design and participants

TRAILBLAZER-ALZ 6 (NCT05738486), a multicenter, randomized, double-blind, phase 3b study in adults with early symptomatic AD and presence of amyloid pathology, as assessed by positron emission tomography (PET) scans, compared the standard donanemab dosing regimen (2) to 3 alternative donanemab dosing arms. A total of 843 participants were randomly assigned in a 1:1:1:1 ratio. Complete study design details, including enrollment and dose stopping criteria, were previously reported [8]. The protocol was approved by local ethical review boards, and the study was conducted in accordance with the ethical standards set forth in the Declaration of Helsinki and all applicable laws and regulations. All participants provided written informed consent before undergoing any study-specific procedures.

### 2.2 Outcomes

The primary outcome in TRAILBLAZER-ALZ 6 was the frequency of ARIA-E at 24 weeks in the alternative donanemab dosing arms compared to the standard donanemab dosing arm.

Secondary outcomes included the effects on brain amyloid levels, frequency of ARIA with hemorrhages/hemosiderin deposition (ARIA-H), and the severity of ARIA-E and ARIA-H.

Plasma phosphorylated tau (P-tau)217 samples were collected and assessed as previously described [8]. Glial fibrillary acidic protein (GFAP) samples were measured using the Simoa® Human Neurology 4-Plex E (N4PE) assay.

### 2.3 Statistical analyses

A Bayesian logistic regression model which included fixed effects for treatment regimen, apolipoprotein E ε4 status, baseline presence of microhemorrhage, baseline presence of cortical superficial siderosis, and baseline amyloid level, was used to evaluate the relative reduction in ARIA frequencies through week 76 for each alternative dosing arm compared to the standard dosing arm. The prespecified success criterion was a posterior probability of more than 80 % that at least 1 alternative dosing arm reduced ARIA risk (RRR) by at least 20 % compared to the standard arm by 24 weeks. ARIA severity was assessed with the Cochran-Mantel-Haenszel test [9].

The effect of each alternative donanemab dosing regimen compared with the standard donanemab dosing regimen on brain amyloid deposition, plasma P-tau217, and plasma GFAP through 76 weeks was assessed using a mixed model for repeated measures (MMRM) with fixed effects for treatment, visit, treatment-by-visit interaction, baseline value, baseline value-by-visit interaction, and baseline age with an unstructured variance–covariance.

*All safety-related outcomes, including the primary endpoint, were based on the safety analysis set (defined as all participants randomly assigned to study treatment who received at least 1 dose of study treatment).*

## 3 Results

The results presented in this report are based on the 76-week database lock date of 03 April 2025.

### 3.1 Participants

Overall, 2529 adults with early symptomatic AD were assessed for eligibility. Of these, 843 were randomly assigned to a donanemab dosing regimen: standard (*N* = 208), modified titration (*N* = 212), dose skipping (*N* = 210) and maximum observed drug concentration (Cmax) (*N* = 213) arm. The demographic and baseline characteristics of participants were balanced across arms as previously described [8].

### 3.2 ARIA-E

By 76 weeks, 15.6 %, 18.6 %, and 19.2 % of participants experienced ARIA-E in the modified titration, dose skipping, and Cmax arms, respectively, compared to 24.2 % in the standard arm. The modified titration arm had an 87 % (>80 %) probability of achieving ≥20 % RRR in ARIA-E frequency compared to the standard arm, and the posterior RRR (standard deviation) was 34.6 % (12.8 %) (Table 1). Although data from all arms are shown for reference, this report focuses on the modified titration arm—the only arm that met the primary endpoint.

**Table 1:** TRAILBLAZER-ALZ 6 study results through 76 weeks.

Category	Standard (*N* = 207)	Modified titration (*N* = 212)	Dose skipping (*N* = 210)	Cmax (*N* = 213)
**Safety overview,** n ( %)				
Deaths	1 (0.5)	1 (0.5)	1 (0.5)	2 (0.9)
SAEs	51 (24.6)	49 (23.1)	42 (20.0)	38 (17.8)
Discontinuations from study due to an AE	6 (2.9)	6 (2.8)	7 (3.3)	9 (4.2)
Discontinuations from study treatment due to an AE	16 (7.7)	17 (8.0)	22 (10.5)	25 (11.7)
TEAEs	191 (92.3)	199 (93.9)	195 (92.9)	189 (88.7)
TEAEs related to study treatment	110 (53.1)	114 (53.8)	102 (48.6)	118 (55.4)
**ARIA-E**^a^^,^^b^	50 (24.2)	33 (15.6)	39 (18.6)	41 (19.2)
Symptomatic^a^^,^^b^, n ( %)	10 (4.8)	6 (2.8)	11 (5.2)	11 (5.2)
SAE of ARIA-E^c^, n ( %)	0	1 (0.5)	4 (1.9)	2 (0.9)
ARIA-oedema/effusion^c^, n ( %)	0	1 (0.5)	4 (1.9)	2 (0.9)
RRR vs standard arm^a^^†^				
Posterior RRR (SD)	—	0.346 (0.128)	0.215 (0.146)	0.190 (0.144)
95 % CrI	—	0.061, 0.565	−0.104, 0.470	−0.128, 0.438
Posterior probability of RRR ≥20 %	—	0.871*	0.574	0.505
Maximum radiographic severity^d^, n ( %)				
Not present	157 (75.8)	179 (84.4)	173 (82.4)	172 (80.8)
Mild	17 (8.2)	13 (6.1)	10 (4.8)	15 (7.0)
Moderate	29 (14.0)	20 (9.4)	23 (11.0)	22 (10.3)
Severe	4 (1.9)	0	4 (1.9)	4 (1.9)
P-value versus standard arm	—	0.015	n.s	n.s
*APOE* ɛ4 genotype^d^, N; n ( %)				
Homozygous	21; 12 (57.1)	21; 5 (23.8)	22; 8 (36.4)	21; 9 (42.9)
Heterozygous	112; 27 (24.1)	115; 18 (15.7)	115; 22 (19.1)	116; 25 (21.6)
Non-carrier	72; 11 (15.3)	75; 10 (13.3)	73; 7 (9.6)	76; 7 (9.2)
Discontinuations due to ARIA-E	2 (1.0)	3 (1.4)	5 (2.4)	4 (1.9)
**ARIA-H**^a^^,^^e^	57 (27.5)	54 (25.5)	59 (28.1)	62 (29.1)
Symptomatic^a^^,^^e^^,^^f^, n ( %)	1 (0.5)	1 (0.5)	1 (0.5)	3 (1.4)
SAE of ARIA-H^c^, n ( %)	0	0	0	0
RRR vs standard arm^a^^,^‡				
Posterior RRR (SD)		0.065 (0.143)	−0.032 (0.152)	−0.068 (0.158)
95 % CrI		−0.243, 0.319	−0.371, 0.234	−0.405, 0.214
Posterior Probability of RRR ≥20 %		0.173	0.047	0.031
Maximum Radiographic Severity^d^, n ( %)				
Not present	152 (73.4)	158 (74.5)	152 (72.4)	152 (71.4)
Mild	29 (14.0)	36 (17.0)	31 (14.8)	32 (15.0)
Moderate	13 (6.3)	9 (4.2)	9 (4.3)	16 (7.5)
Severe	13 (6.3)	9 (4.2)	18 (8.6)	13 (6.1)
*APOE* ɛ4 genotype^d^, N; n ( %)				
Homozygous	21; 10 (47.6)	21; 6 (28.6)	22; 13 (59.1)	21; 13 (61.9)
Heterozygous	112; 35 (31.3)	115; 33 (28.7)	115; 35 (30.4)	116; 31 (26.7)
Non-carrier	72; 10 (13.9)	75; 15 (20.0)	73; 10 (13.7)	76; 17 (22.4)
Microhemorrhage^d^, n ( %)	45 (21.7)	49 (23.1)	52 (24.8)	54 (25.4)
RRR vs standard arm^d^^,^§				
Posterior RRR (SD)	—	−0.068 (0.186)	−0.146 (0.194)	−0.169 (0.193)
95 % CrI	—	−0.478, 0.246	−0.567, 0.181	−0.590, 0.162
Posterior probability of RRR ≥20 %	—	0.053	0.019	0.013
Maximum radiographic severity^d^, n ( %)				
Not present	162 (78.3)	163 (76.9)	158 (75.2)	159 (74.6)
Mild	27 (13.0)	38 (17.9)	30 (14.3)	33 (15.5)
Moderate	12 (5.8)	6 (2.8)	10 (4.8)	9 (4.2)
Severe	6 (2.9)	5 (2.4)	12 (5.7)	12 (5.6)
Cortical superficial siderosis^d^, n ( %)	31 (15.0)	19 (9.0)	20 (9.5)	27 (12.7)
Relative risk reduction vs standard arm^f^^,^¶				
Posterior RRR (SD)	—	0.388 (0.160)	0.349 (0.167)	0.136 (0.198)
95 % CrI	—	0.028, 0.648	−0.028, 0.623	−0.305, 0.464
Posterior Probability of RRR ≥20 %	—	0.877*	0.826*	0.412
Maximum Radiographic Severity^d^, n ( %)				
Not present	176 (85.0)	193 (91.0)	190 (90.5)	186 (87.3)
Mild	18 (8.7)	10 (4.7)	4 (1.9)	14 (6.6)
Moderate	4 (1.9)	5 (2.4)	3 (1.4)	8 (3.8)
Severe	9 (4.3)	4 (1.9)	13 (6.2)	5 (2.3)
Discontinuations due to ARIA-H	2 (1.0)	2 (0.9)	2 (1.0)	1 (0.5)
**Macrohemorrhages**^a^**^,^**^g^**, n ( %)**	1 (0.5)	2 (0.9)	1 (0.5)	2 (0.9)
SAE of macrohemorrhage^c^, n ( %)	0	1 (0.5)	0	0
Cerebral hemorrhage^c^, n ( %)	0	1 (0.5)	0	0
**Any ARIA (either E or H)**^a^**^,^**^b^**^,^**^e^, n ( %)	71 (34.3)	61 (28.8)	68 (32.4)	71 (33.3)
Any SAE of ARIA (either E or H)^c^, n ( %)	0 (0)	1 (0.5)	4 (1.9)	2 (0.9)
RRR vs standard arm^a^^,^#				
Posterior RRR (SD)	—	0.146 (0.120)	0.042 (0.126)	0.014 (0.128)
95 % CrI	—	−0.113, 0.356	−0.223, 0.269	−0.260, 0.243
Posterior probability of RRR ≥20 %	—	0.347	0.096	0.060
**Concurrent ARIA-E and ARIA-H**^d^, n ( %)	34 (16.4)	24 (11.3)	27 (12.9)	31 (14.6)
RRR vs standard arm^f^^,^||				
Posterior RRR (SD)	—	0.294 (0.169)	0.196 (0.178)	0.095 (0.193)
95 % CrI	—	−0.094, 0.574	−0.206, 0.496	−0.344, 0.411
Posterior Probability of RRR ≥20 %	—	0.746	0.539	0.316

a Based on MRI or TEAE cluster.

b ARIA-E TEAE cluster preferred terms are amyloid-related imaging abnormalities oedema/effusion, brain oedema, and vasogenic cerebral oedema.

c Based on TEAE cluster.

d Based on MRI only.

e ARIA-H TEAE cluster preferred terms are ARIA-microhemorrhage and hemosiderin deposits, brainstem microhemorrhage, cerebellar microhemorrhage, cerebral hemosiderin deposit, cerebral microhemorrhage, and cortical superficial siderosis of the central nervous system.

f Symptomatic ARIA-H low level term includes symptomatic ARIA-H, symptomatic ARIA-microhemorrhages and haemosiderin deposits, symptomatic ARIA-microhemorrhages and hemosiderin deposits, and symptomatic ARIA-cortical superficial siderosis.

g Macrohemorrhage preferred terms are cerebral hemorrhage and hemorrhagic stroke.

⁎ Significant vs standard arm. Intercept prior was elicited as *N*= ^†^(−1.40, 8.32).

‡ (−0.98, 9.66).

§ (−1.24, 8.75).

¶ (−1.97, 7.24).

# (−0.75, 10.74).

|| (−1.99, 7.21). Abbreviations: AE, adverse event; APOE, apolipoprotein E; ARIA, amyloid-related imaging abnormalities; ARIA-E, amyloid-related imaging abnormalities with edema/effusions; ARIA-H, amyloid-related imaging abnormalities with hemorrhages/hemosiderin deposition; Cmax, maximum observed drug concentration; Crl, credible interval; MRI, magnetic resonance imaging; n.s., nonsignificant; RRR, relative risk reduction; SAE, serious adverse event; SD, standard deviation; TEAE, treatment-emergent adverse event.

The modified titration arm also improved ARIA-E severity at 76 weeks (*p* = 0.015), with no radiographically severe events observed through 76 weeks. Notably, 84.4 % of participants in the modified titration arm had no ARIA-E through week 76 compared to 75.8 % in the standard arm.

**Fig. 1:**
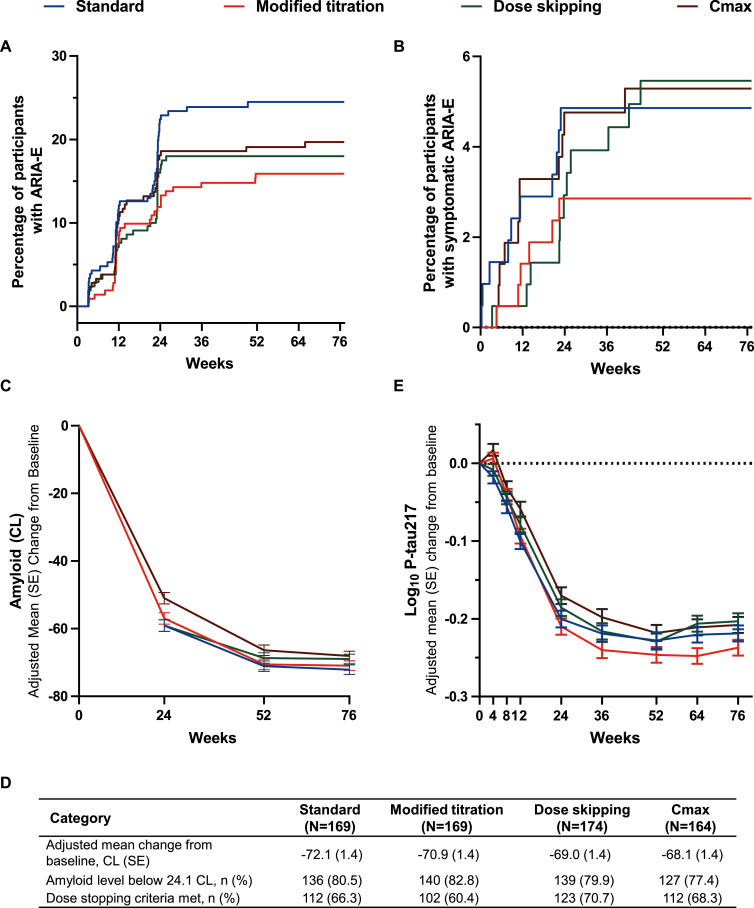
**The modified titration arm significantly reduced ARIA-E risk while demonstrating comparable pharmacodynamics to standard dosing through 76 weeks.** (A), (B) Kaplan-Meier curves showing time to first event based on magnetic resonance imaging only. Participants at risk are the number of participants still active in the study who have not yet experienced the event. (A) There is a significant (log-rank unstratified [2-sided] *p* = 0.028) reduction of risk with the modified titration arm compared to the standard arm. (B) Although the modified titration arm shows a decrease in symptomatic ARIA-E risk, it is not significant compared to the standard arm. (C), (D) Reductions in amyloid level, the percent of participants with an amyloid level below the 24.1 CL threshold, and the percent of participants who met dose stopping criteria were similar between the modified titration and standard arms. (E) Plasma P-tau217 reduction in the modified titration and standard arms were similar. Abbreviations: ARIA-E, amyloid-related imaging abnormalities with edema/effusions; CL, Centiloid; CMAX, maximum observed drug concentration; n, number of participants within each specific category; P-tau, phosphorylated tau; SE, standard error.

Cox proportional hazard analysis of time to first ARIA-E showed that the modified titration arm had a significantly lower percentage of participants with ARIA-E risk (*p* = 0.028) (Fig. 1A). Modified titration had a non-significant but numerically lower percentage of symptomatic ARIA-E (*p* = 0.288) (Fig. 1B) compared to the standard dosing arm through 76 weeks. All initial symptomatic ARIA-E occurred within the first 24 weeks in the modified titration and standard arms.

### 3.3 ARIA-H and macrohemorrhage

By 76 weeks, 25.5 % of participants in the modified titration arm and 27.5 % of participants in the standard arm had experienced ARIA-H (Table 1). The frequency of microhemorrhage was not significantly different in the modified titration arm compared with the standard arm. In contrast, cortical superficial siderosis was significantly reduced in the modified titration arm (9.0 % of participants) compared with the standard arm (15.0 % of participants), with a 38.8 % RRR and an 87.7 % probability that the RRR was ≥20 % (Table 1). The frequencies of concurrent ARIA-E and ARIA-H events were 11.3 % and 16.4 % of participants in the modified titration and standard arms, respectively. The radiographic severity of ARIA-H was not significantly different between the standard and modified titrations arms (Table 1).

Macrohemorrhage occurred in 2 participants (0.9 %) in the modified titration arm and 1 participant (0.5 %) in the standard arm (Table 1) (all of which occurred by week 24).

### 3.4 Additional safety outcomes

There was 1 death (0.5 %) in the modified titration arm and 1 death (0.5 %) in the standard arm during the overall 76-week treatment period (Table 1). These deaths occurred by week 52 and were previously reported [8]. Briefly, the death in the modified titration arm occurred within the first 24 weeks and was due to cerebral hemorrhage after administration of tissue plasminogen activator for presumed right middle cerebral artery stroke. The death in the standard arm occurred between 24 and 52 weeks and was due to cardio-respiratory arrest, which occurred approximately 3 months after meeting dose stopping criteria. The frequency of serious adverse events was 23.1 % in the modified titration arm and 24.6 % in the standard arm. The frequency of treatment-emergent adverse events was also similar in both arms (93.9 % in the modified titration arm compared to 92.3 % in the standard arm) (Table 1). Treatment-emergent adverse events reported by more than 15 % of participants in the modified titration arm (versus the standard arm) were headache (20.3 % vs 24.2 %), fall (19.8 % vs 14.0 %), ARIA-H (18.4 % vs 19.8 %), COVID-19 (17.9 % vs 12.6 %), infusion related reaction (17.9 % vs 14.0 %) and ARIA-E (15.6 % vs 24.2 %). Discontinuations due to adverse events were similar in the modified titration and standard arms (Table 1).

### 3.5 Biomarkers

The adjusted mean change (standard error) in amyloid from baseline to 76 weeks was −70.9 (1.4) Centiloids (CL) in the modified titration arm and −72.1 (1.4) CL in the standard arm (Fig. 1C and 1D). The change in amyloid levels from baseline to week 76 was comparable in both treatment arms, and the difference between the two arms was not significant. An amyloid threshold level below 24.1 CL was reached by 82.8 % and 80.5 % of participants in the modified titration and standard arms, respectively (Fig. 1D). The amyloid PET criteria for donanemab dose cessation [8] was met by 60.4 % of participants in the modified titration arm and 66.3 % in the standard arm at week 76 (Fig. 1D). Plasma phosphorylated tau (P-tau)217, assessed as an exploratory objective, was similarly reduced in both the modified titration and standard arms (Fig. 1E). Plasma GFAP levels were also assessed as an exploratory objective and comparable reductions were seen between the two arms (Table 2).

**Table 2:** Key TRAILBLAZER-ALZ 6 study results at 24, 52 and 76 weeks.

Category	Standard (*N* = 207)	Modified titration (*N* = 212)	Dose skipping (*N* = 210)	Cmax (*N* = 213)
**ARIA-E**^a^**^,^**^b^				
24 weeks	49 (23.7)	29 (13.7)	39 (18.6)	39 (18.3)
52 weeks	50 (24.2)	33 (15.6)	39 (18.6)	40 (18.8)
76 weeks	50 (24.2)	33 (15.6)	39 (18.6)	41 (19.2)
RRR vs standard arm^a^				
24 weeks ^†^				
Posterior RRR (SD) 95 % CrI	—	0.405 (0.123) 0.135, 0.616	0.195 (0.146) −0.130, 0.447	0.211(0.145) −0.097, 0.465
Posterior probability of RRR ≥20 %	—	94.1*	51.2	56.6
52 weeks‡				
Posterior RRR (SD) 95 % CrI	—	0.347 (0.129) 0.065, 0.569	0.218 (0.147) −0.105, 0.472	0.209 (0.144) −0.101, 0.455
Posterior probability of RRR ≥20 %	—	87.0*	58.4	55.7
76 weeks§				
Posterior RRR (SD) 95 % CrI	—	0.346 (0.128) 0.061, 0.565	0.215 (0.146) −0.104, 0.470	0.190 (0.144) −0.128, 0.438
Posterior probability of RRR ≥20 %	—	87.1*	57.4	50.5
SAE of ARIA-E^c^, n ( %)				
24 weeks	0 (0)	0 (0)	0 (0)	1 (0.5)
52 weeks	0 (0)	1 (0.5)	4 (1.9)	2 (0.9)
76 weeks	0 (0)	1 (0.5)	4 (1.9)	2 (0.9)
Symptomatic^a^^,^^b^, n ( %)				
24 weeks	10 (4.8)	6 (2.8)	8 (3.8)	11 (5.2)
52 weeks	10 (4.8)	6 (2.8)	11 (5.2)	11 (5.2)
76 weeks	10 (4.8)	6 (2.8)	11 (5.2)	11 (5.2)
Maximum radiographic severity^d^, n ( %)				
24 weeks				
Not present	158 (76.3)	183 (86.3)	173 (82.4)	174 (81.7)
Mild	19 (9.2)	10 (4.7)	11 (5.2)	15 (7.0)
Moderate	26 (12.6)	19 (9.0)	22 (10.5)	21 (9.9)
Severe	4 (1.9)	0 (0)	4 (1.9)	3 (1.4)
P-value versus standard arm	—	0.011*	0.271	0.210
52 weeks				
Not present	157 (75.8)	179 (84.4)	173 (82.4)	173 (81.2)
Mild	17 (8.2)	13 (6.1)	10 (4.8)	14 (6.6)
Moderate	29 (14.0)	20 (9.4)	23 (11.0)	22 (10.3)
Severe	4 (1.9)	0 (0)	4 (1.9)	4 (1.9)
P-value versus standard arm	—	0.015*	0.203	0.223
76 weeks				
Not present	157 (75.8)	179 (84.4)	173 (82.4)	172 (80.8)
Mild	17 (8.2)	13 (6.1)	10 (4.8)	15 (7.0)
Moderate	29 (14.0)	20 (9.4)	23 (11.0)	22 (10.3)
Severe	4 (1.9)	0	4 (1.9)	4 (1.9)
P-value versus standard arm	—	0.015*	0.203	0.247
**ARIA-H**^a^**^,^**^e^				
24 weeks	52 (25.1)	43 (20.3)	48 (22.9)	44 (20.7)
52 weeks	57 (27.5)	53 (25.0)	59 (28.1)	60 (28.2)
76 weeks	57 (27.5)	54 (25.5)	59 (28.1)	62 (29.1)
Symptomatic^a^^,^^e^^,^^f^, n ( %)				
24 weeks	0 (0)	1 (0.5)	0 (0)	4 (1.9)
52 weeks	0 (0)	1 (0.5)	1 (0.5)	3 (1.4)
76 weeks	1 (0.5)	1 (0.5)	1 (0.5)	3 (1.4)
SAE of ARIA-H^c^, n ( %)				
24 weeks	0 (0)	0 (0)	0 (0)	0 (0)
52 weeks	0 (0)	0 (0)	0 (0)	0 (0)
76 weeks	0 (0)	0 (0)	0 (0)	0 (0)
Maximum radiographic severity^d^, n ( %)				
24 weeks				
Not present	156 (75.4)	169 (79.7)	163 (77.6)	169 (79.3)
Mild	34 (16.4)	31 (14.6)	26 (12.4)	26 (12.2)
Moderate	12 (5.8)	9 (4.2)	7 (3.3)	8 (3.8)
Severe	5 (2.4)	3 (1.4)	14 (6.7)	10 (4.7)
52 weeks				
Not present	152 (73.4)	159 (75.0)	152 (72.4)	153 (71.8)
Mild	30 (14.5)	35 (16.5)	32 (15.2)	31 (14.6)
Moderate	12 (5.8)	10 (4.7)	8 (3.8)	17 (8.0)
Severe	13 (6.3)	8 (3.8)	18 (8.6)	12 (5.6)
76 weeks				
Not present	162 (78.3)	163 (76.9)	152 (72.4)	152 (71.4)
Mild	27 (13.0)	38 (17.9)	31 (14.8)	32 (15.0)
Moderate	12 (5.8)	6 (2.8)	9 (4.3)	16 (7.5)
Severe	6 (2.9)	5 (2.4)	18 (8.6)	13 (6.1)
**Macrohemorrhages**^a^**^,^**^g^**, n ( %)**				
24 weeks	1 (0.5)	2 (0.9)	0 (0)	1 (0.5)
52 weeks	1 (0.5)	2 (0.9)	1 (0.5)	1 (0.5)
76 weeks	1 (0.5)	2 (0.9)	1 (0.5)	2 (0.9)
SAE of macrohemorrhage^c^, n ( %)				
24 weeks	0 (0)	1 (0.5)	0 (0)	0 (0)
52 weeks	0 (0)	1 (0.5)	0 (0)	0 (0)
76 weeks	0 (0)	1 (0.5)	0 (0)	0 (0)
** Amyloid adjusted mean change from baseline, CL (SE)**				
24 weeks	−59.1 (1.74)	−57.0 (1.71)	−59.1 (1.719)	−51.0 (1.718)
52 weeks	−71.1 (1.56)	−70.6 (1.55)	−68.7 (1.540)	−66.4 (1.555)
76 weeks	−72.1 (1.42)	−70.9 (1.41)	−69.0 (1.398)	−68.1 (1.420)
** Plasma P-tau217 (log_10_ [pg/ml]) adjusted mean change from baseline (SE)**				
24 weeks	−0.20 (0.011)	−0.21 (0.011)	−0.19 (0.011)	−0.17 (0.011)
52 weeks	−0.23 (0.010)	−0.25 (0.010)	−0.23 (0.010)	−0.22 (0.010)
76 weeks	−0.22 (0.010)	−0.24 (0.010)	−0.20 (0.010)	−0.21 (0.010)
** Plasma GFAP (log_10_ [pg/ml]) adjusted mean change from baseline (SE)**				
24 weeks	−0.06 (0.010)	−0.07 (0.010)	−0.05 (0.010)	−0.06 (0.010)
52 weeks	−0.09 (0.010)	−0.10 (0.010)	−0.08 (0.010)	−0.09 (0.010)
76 weeks	−0.08 (0.011)	−0.09 (0.011)	−0.09 (0.011)	−0.08 (0.011)

a Based on MRI or TEAE cluster.

b ARIA-E TEAE cluster preferred terms are amyloid-related imaging abnormalities oedema/effusion, brain oedema, and vasogenic cerebral oedema.

c Based on TEAE cluster.

d Based on MRI only.

e ARIA-H TEAE cluster preferred terms are ARIA-microhemorrhage and hemosiderin deposits, brainstem microhemorrhage, cerebellar microhemorrhage, cerebral hemosiderin deposit, cerebral microhemorrhage, and cortical superficial siderosis of the central nervous system.

f Symptomatic ARIA-H low level term includes symptomatic ARIA-H, symptomatic ARIA-microhemorrhages and haemosiderin deposits, symptomatic ARIA-microhemorrhages and hemosiderin deposits, and symptomatic ARIA-cortical superficial siderosis.

g Macrohemorrhage preferred terms are cerebral hemorrhage and hemorrhagic stroke.

⁎ Significant vs standard arm. Intercept prior was elicited as *N*= ^†^(−1.49, 8.10).

‡ (−1.42, 8.27).

§ (−1.40, 8.32). Abbreviations: ARIA, amyloid-related imaging abnormalities; ARIA-E, amyloid-related imaging abnormalities with edema/effusions; ARIA-H, amyloid-related imaging abnormalities with hemorrhages/hemosiderin deposition; CL, Centiloids, Cmax, maximum observed drug concentration; Crl, credible interval; GFAP, Glial fibrillary acidic protein; MRI, magnetic resonance imaging; P-tau217, Plasma phosphorylated tau (P-tau)217; RRR, relative risk reduction; SAE, serious adverse event; SD, standard deviation; SE, standard error; TEAE, treatment-emergent adverse event.

## 4 Discussion

The primary endpoint for this study, reduced frequency of ARIA-E at 24 weeks with an alternative donanemab dosing regimen compared with the standard donanemab dosing regimen, was met with the modified titration arm as previously reported [8]. The dosing difference between the modified titration arm and the standard arm was the timing of a single vial (350 mg), which was removed from the first infusion and added to the third infusion.

Both the 52- and 76-week results supported the 24-week data with a lower frequency of ARIA-E in the modified titration arm compared to the standard arm (Table 2). The underlying mechanism for this result remains unknown; however, as discussed previously [8], the initial lower dose of donanemab in the modified titration arm may enable more gradual amyloid mobilization, thereby reducing inflammation and vascular permeability, which could contribute to a lower ARIA-E risk. The absence of new ARIA-E events in both the modified titration and standard arms after 52 weeks supports that ARIA-E was reduced rather than delayed in the modified titration arm in which approximately 90 % of ARIA-E occurred within the first 6 months.

Importantly, participants in the modified titration and standard arms had a similar amyloid reduction from baseline as assessed by PET scans (adjusted mean change at 76 weeks: 70.9 CL versus 72.1 CL, respectively). Participants in the modified titration and standard arms also had a similar P-tau217 response.

Some study limitations should be noted. The study size limits the ability to detect significant differences in small subgroups or less frequent safety events. Furthermore, the study was conducted in 2 countries, representing a small geographic scope. Lastly, TRAILBLAZER-ALZ 6 was designed as a safety study, so clinical changes in cognition and function were not assessed. However, amyloid reduction, which was similar in both the modified titration and standard arms, has been shown to be associated with clinical benefits [10,11].

The goal of TRAILBLAZER-ALZ 6 was to determine if the risks of amyloid-targeting therapies for patients with early symptomatic AD could be lowered with alternative dosing regimens. This study showed that gradual titration of donanemab can decrease the frequency of ARIA-E without impacting amyloid removal.

## Funding sources

Eli Lilly and Company.

## Declaration of generative AI and AI-assisted technologies in the writing process

No AI was used in the writing process.

## Consent statement

All participants provided written informed consent before undergoing any study-specific procedures.

## CRediT authorship contribution statement

**Hong Wang:** Writing – review & editing, Methodology, Investigation. **Emel Serap Monkul Nery:** Writing – review & editing, Methodology, Investigation. **Paul Ardayfio:** Writing – review & editing, Methodology. **Rashna Khanna:** Writing – review & editing, Conceptualization. **Diana Otero Svaldi:** Writing – review & editing, Methodology, Investigation. **Sergey Shcherbinin:** Writing – review & editing. **Wen Xu:** Writing – review & editing, Validation, Methodology, Conceptualization. **Scott W. Andersen:** Writing – review & editing, Formal analysis. **Paula M. Hauck:** Writing – review & editing, Writing – original draft, Visualization. **Dawn A. Brooks:** Writing – review & editing, Conceptualization. **Emily C. Collins:** Writing – review & editing, Methodology, Investigation. **Stephen Salloway:** Writing – review & editing. **Mark A. Mintun:** Writing – review & editing, Methodology, Conceptualization. **John R. Sims:** Writing – review & editing, Methodology, Conceptualization.

## Declaration of competing interest

The authors declare the following financial interests/personal relationships which may be considered as potential competing interests:

Stephen Salloway reports a relationship with Biogen that includes: consulting or advisory and funding grants. Stephen Salloway reports a relationship with Eisai that includes: consulting or advisory and funding grants. Stephen Salloway reports a relationship with Genentech that includes: consulting or advisory and funding grants. Stephen Salloway reports a relationship with Roche that includes: consulting or advisory and funding grants. Stephen Salloway reports a relationship with Cognition that includes: consulting or advisory and funding grants. Stephen Salloway reports a relationship with Eli Lilly and Company that includes: consulting or advisory and funding grants. Stephen Salloway reports a relationship with Janssen that includes: funding grants. Stephen Salloway reports a relationship with Abbvie that includes: consulting or advisory. Stephen Salloway reports a relationship with Acumen that includes: consulting or advisory. Stephen Salloway reports a relationship with Alector that includes: consulting or advisory. Stephen Salloway reports a relationship with Biohaven that includes: consulting or advisory. Stephen Salloway reports a relationship with Fujirebio that includes: consulting or advisory. Stephen Salloway reports a relationship with Kisbee that includes: consulting or advisory. Stephen Salloway reports a relationship with LABCORP that includes: consulting or advisory. Stephen Salloway reports a relationship with Merck that includes: consulting or advisory. Stephen Salloway reports a relationship with Neurophet that includes: consulting or advisory. Stephen Salloway reports a relationship with NovoNordisk that includes: consulting or advisory. Stephen Salloway reports a relationship with Prothena that includes: consulting or advisory. Stephen Salloway reports a relationship with Quest that includes: consulting or advisory. If there are other authors, they declare that they have no known competing financial interests or personal relationships that could have appeared to influence the work reported in this paper.
